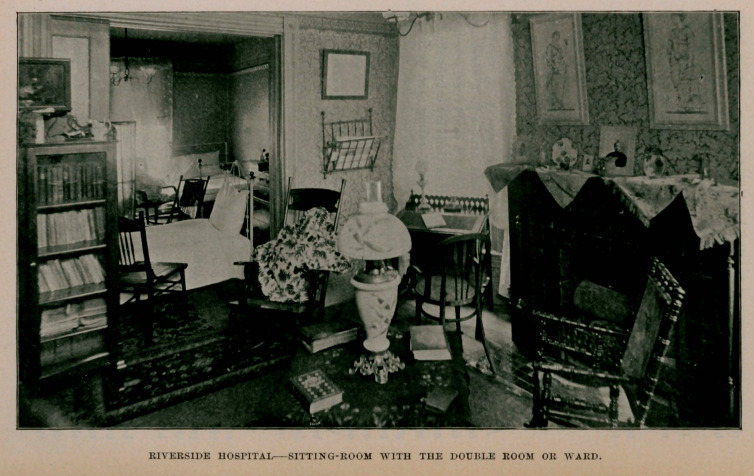# Riverside Hospital

**Published:** 1896-06

**Authors:** Lillian Craig Randall

**Affiliations:** Buffalo, N.Y.; 502 Elmwood Avenue


### RIVERSIDE HOSPITAL.

By LILLIAN CRAIG RANDALL, M. D., Buffalo, N. Y.

A VARIETY of motives led to the establishment of this hospital in 1892, by Dr. Lillian Craig Randall and Dr. Mary T. Green.

By way of note, the most important was the opinion frequently expressed by brother physicians as well as by women patients, that a woman as resident physician in our hospitals would help to obviate many of the disagreeable features connected with the preparation and after-care of operative gynecology; again, certain surgical experience could only be obtained in Buffalo by women through such a procedure.

These and other motives led to the opening of such a private hospital. Its steady, healthy growth is the reason for its continued existence.

It is thought to be the only hospital controlled and owned by a woman physician, where all general hospital work is done, and in this connection it is of interest in this number of the women's edition of the BUFFALO MEDICAL JOURNAL.

In June, 1892, three rooms were secured as temporary quarters at 2002 Niagara street. These rooms were provided with three iron beds and one cot bed for a nurse, the most simple household and hospital appliances, combined with surgical cleanliness.

Case I. Maternity. Sent for care by Dr. Irving M. Snow. A married woman, Canadian. In her two previous confinements she had entered the lying-in department of the Toronto hospital, and although the change was great she left with her baby, well pleased. This case is of especial interest as our first.

Case II. An operation sent by Dr. M. A. Crockett, to whom, as operator and consultant, much of the success which has come to this little hospital is due. Case II. made a good recovery.

The next six months were of slow progress. Thirty-six were treated, nearly all gynecological, all successful, and no more

RIVERSIDE HOSPITAL—OPERATING ROOM.

maternity cases came until January, 1893, although many cases applied for free treatment.

December, 1892. As four patients were to be cared for and space permitted but three hospital beds, it was deemed advisable to enlarge the boundaries. A house on the banks of the Niagara river was secured and eight beds provided. Many hospital appliances were added, and a distinctive name, Riverside Hospital, chosen.

The staff was then appointed and consisted of Drs. Randall and Green as house physicians; Dr. M. A. Crockett, gynecologist; Dr. J. W. Putnam, neurologist, and Dr. I. M. Snow, in charge of diseases of children.

As the project was now under way the doctors in charge took this opportunity to call personally upon the physicians in the vicinity in order to explain the work and to ask for their support and coöperation. In each case the projectors of this hospital scheme were received with unfailing courtesy. In some cases the barely perceptible flicker causing a twinkle of the professional eye was just enough to remind one of the early days and the pioneer work done by Dr. Emily Blackwell when she, with much more at stake, started the New York Infirmary for Women and Children. The success of an undertaking in this end of the century days of competition depends very largely upon the ability to fill some want, and the want in this particular venture was hospital care at reasonable rates, where patients might be by themselves and also in charge of the physician sending in the case, while the hospital would provide trained service, as well as attention from a woman physician.

Like all work which meets with healthy success Riverside has grown slowly, not especially as woman's work, but through the hearty coöperation of doctors without regard to sex. Nor is it in any way an aggressive attempt to show that woman as well as man can do hospital work, but it does claim to stand upon its merits and upon the same grounds as do other institutions doing surgical and medical work.

Soon after the removal to Niagara street, Dr. Green withdrew her interest from Riverside and removed to Pike, N. Y., Dr. Randall continuing the hospital work.

An addition to the staff was made by securing Dr. John C. Thompson and Dr. Julius H. Potter in general medicine. Up to this time Riverside had received only women patients, but the

RIVERSIDE HOSPITAL—PRIVATE ROOM.

demand came for the care of men, and the facilities being enlarged, a resident male student secured, this change was made.

At this time a training school for nurses was organised, a course selected occupying two years, the practical experience at Riverside to be supplemented by attendance at the General Hospital clinics; the Clara Weeks-Shaw text-book chosen, a graduate nurse from the Buffalo General secured as superintendent, and the school with two pupil nurses began lessons in May, 1893. The first graduation of one nurse was in May, 1895.

The present class contains four pupils, and the following course has been given during the year: Surgical cleanliness, Dr. M. A. Crockett; medicine and medical nursing, Dr. Julius H. Potter; obstetrics, Dr. Lillian C. Randall; urinalysis, Dr. John C. Thompson; care of nervous patients, Dr. James W. Putnam; diet and digestion, Dr. John C. Thompson; anatomy, Dr. Lillian C. Randall.

Besides the regular course, lessons supplementary have been given by Dr. Helene Kuhlman. The success of the class in nursing bids fair to continue a feature of the Riverside work in that direction.

In 1894 the hospital was removed to larger quarters near the same place, but after eighteen months it was deemed advisable, as the trolley ambulance system is yet a thing of the future, to move to a more central location. Temporary but hygienic and comfortable quarters were secured at the present home, 327 Breckenridge street, which will be known as Riverside Hospital until a permanent home is decided upon. The hospital can now accommodate fifteen; has a light, large operating room, shown in the illustration, private rooms, one of which is also shown, and also a sitting-room with the double room or ward.

The present staff consists of: Physician in charge, Lillian Craig Randall, M. D. Attending physicians, Montgomery A. Crockett, M. D., gynecologist; James W. Putnam, M. D., neurologist; John Parmenter, M. D., surgeon; Julius H. Potter, M. D., John C. Thompson, M. D., general medicine. Consultants, Charles G. Stockton, M. D., Electa B. Whipple, M. D., Irving M. Snow, M. D., Grover W. Wende, M. D., Bernard Bartow, M. D. House physician, John J. Cullinane, M. D.

In one way Riverside is an exception to most institutions: it has been self-supporting from the first.

The number of operative cases have been many, and it is wor-

RIVERSIDE HOSPITAL—SITTING-ROOM WITH THE DOUBLE ROOM OR WARD.

thy of note that out of 160 cases but one, a case brought to the hospital when in extremis from appendicitis, was lost.

Nervous patients who come for treatment are under the care of Dr. James W. Putnam, and baths, electricity and massage are employed for these special cases.

The belief that the sick public is making use of the advantages offered by hospital care more and more each year offers a future hopeful to hospital work generally.

502 ELMWOOD AVENUE.

## Figures and Tables

**Figure f1:**
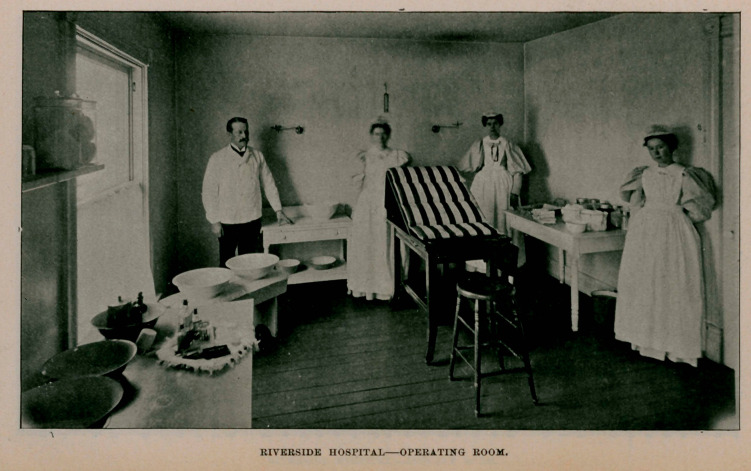


**Figure f2:**
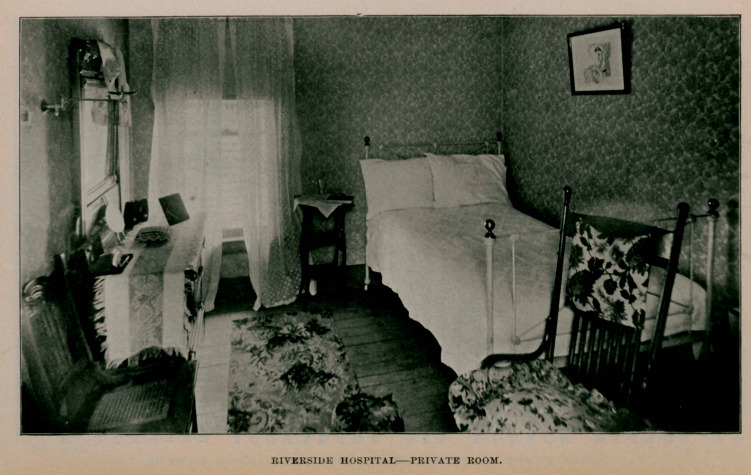


**Figure f3:**